# Nano Hard Carbon Anodes for Sodium-Ion Batteries

**DOI:** 10.3390/nano9050793

**Published:** 2019-05-23

**Authors:** Dae-Yeong Kim, Dong-Hyun Kim, Soo-Hyun Kim, Eun-Kyung Lee, Sang-Kyun Park, Ji-Woong Lee, Yong-Sup Yun, Si-Young Choi, Jun Kang

**Affiliations:** 1 Division of Marine Engineering, Korea Maritime and Ocean University, 727 Taejong-ro, Yeongdo-gu, Busan 49112, Korea; smap1211@kmou.ac.kr; 2 Korea Maritime Equipment Research Institute/ICT Convergence Team, 435 Haeyang-ro, Yeongdo-gu, Busan 49111, Korea; kdh9942@komeri.re.kr (D.-H.K.); shkim@komeri.re.kr (S.-H.K.); 3 Department of Ocean Advanced Materials Convergence Engineering, Korea Maritime and Ocean University, 727 Taejong-ro, Yeongdo-gu, Busan 49112, Korea; elee@kmou.ac.kr; 4 Division of Marine Information Technology, Korea Maritime and Ocean University, 727 Taejong-ro, Yeongdo-gu, Busan 49112, Korea; skpark@kmou.ac.kr (S.-K.P.); woongsengine@kmou.ac.kr (J.-W.L.); 5 Division of Marine System Engineering, Korea Maritime and Ocean University, 727 Taejong-ro, Yeongdo-gu, Busan 49112, Korea; ysyun@kmou.ac.kr; 6 Department of Materials Science and Engineering, POSTECH, 77 Cheongam-ro, Pohang 37673, Korea

**Keywords:** nano hard carbon, turbostratic structure, solid-electrolyte interphase, co-intercalation reaction, sodium-ion battery

## Abstract

A hindrance to the practical use of sodium-ion batteries is the lack of adequate anode materials. By utilizing the co-intercalation reaction, graphite, which is the most common anode material of lithium-ion batteries, was used for storing sodium ion. However, its performance, such as reversible capacity and coulombic efficiency, remains unsatisfactory for practical needs. Therefore, to overcome these drawbacks, a new carbon material was synthesized so that co-intercalation could occur efficiently. This carbon material has the same morphology as carbon black; that is, it has a wide pathway due to a turbostratic structure, and a short pathway due to small primary particles that allows the co-intercalation reaction to occur efficiently. Additionally, due to the numerous voids present in the inner amorphous structure, the sodium storage capacity was greatly increased. Furthermore, owing to the coarse co-intercalation reaction due to the surface pore structure, the formation of solid-electrolyte interphase was greatly suppressed and the first cycle coulombic efficiency reached 80%. This study shows that the carbon material alone can be used to design good electrode materials for sodium-ion batteries without the use of next-generation materials.

## 1. Introduction

Graphite is the most widely used anode material in lithium-ion batteries (LIBs) owing to its rich reserves, good electrical conductivity, low charge/discharge potential, and excellent cycling stability. Unfortunately, the electrochemical performance of graphite as an anode material is poor in sodium-ion batteries (SIBs) using a conventional carbonate electrolyte. This is due to the passivation layer formed on the graphite surface being less effective than the protective layer formed in a LIB, and due to the continuous decomposition of electrolytes [1]. In addition, due to the thermodynamic instability of sodium, only a small amount of sodium can be stored in graphite. The sodium storage capacity of graphite in a SIB is approximately 1/10th of the lithium storage capacity in a LIB. The reason for this low reversible capacity is not simply the larger ionic radius of Na than that of Li (K is stored more in spite of its larger ionic radius than that of Na). It has been reported that sodium-graphite intercalation compounds (Na-GICs) are more thermodynamically unstable than other alkali-GICs.

In particular, it is estimated that there are three reasons for this instability [2,3,4]. First, the binding energy of GICs are composed of, not only the ion-binding component, but also the covalent bond between the metal and the carbon atom [2]. That is, in the case of alkali metals, as the atomic number increases, the electronegativity decreases, and the alkali-graphite bond stabilizes. In the case of lithium, the covalent contribution of lithium-graphite bonds further stabilizes the bonding state, even if the atom is small. However, sodium has no covalent contribution, and its atomic diameter is smaller than that of other alkali elements, making it more unstable. Next, among the energies required for the M-GIC formation process (energy *E*_d_ required for the alkaline element to detach from the bulk state, energy *E*_g_ necessary to strain the graphite, and energy *E*_b_ reduced by inserting the M atom into the expanded graphite, *E*_f_ = *E*_d_ + *E*_g_ + *E*_b_), the instability is seen as a result of the weakening of *E*_b_. In other words, sodium is the most weakly bonded to the substrate, which is caused by competition between the ionization energy trend below the column of the periodic table and the ion-substrate coupling energy [3]. Finally, the local repulsive interaction between the Na ion and the graphene layer predominantly destabilizes Na-GICs [4]. Therefore, co-intercalation reaction, which promotes the intercalation of Na in graphite by preventing direct interaction between Na and graphene by screening Na with ether solvent molecules, is proposed as an effective strategy [5]. In recent years, carbonate-based electrolytes have been replaced by ether-based electrolytes, and the use of ether-based co-intercalation reactions have been reported to dramatically increase the reversible capacity of graphite [5,6,7]. However, it is still unsatisfactory for practical needs, and the coulombic efficiency (C.E.) varies regardless of the specific surface area.

Therefore, a new carbon material for SIBs was designed to increase the reversible capacity considerably and bring the C.E. to the practical level. The surface area in this study (at least for C.E.) has not been considered because previous studies on storing sodium ions via the co-intercalation reaction have revealed the following results. First, the formation of the solid-electrolyte interphase (SEI) has no relation to the magnitude of specific surface area, and in some cases, no SEI is formed at all. In addition, a very thin, strong SEI has been reported to form [5,7,8,9,10]. Therefore, it was concluded that the larger the specific surface area (that is, the surface pore structure is well developed), the more effective co-intercalation reaction can be generated after the electrolyte forms a complex with sodium without interfering with the pore. It was further concluded that SEI formation could be suppressed and the first cycle C.E. could be greatly improved. An attempt was made to increase the reversible capacity by creating many voids in which sodium could be stored.

It was found that a carbonaceous material with a large storage space is the most suitable because it has a large specific surface area, a wide interlayer distance due to the turbostratic structure, a smooth capacitive reaction, and many internal voids. It was considered that the optimum structure which can have this feature is the carbon black structure. That is, as the primary particles are small, the diffusion distance is short and the specific surface area is large, a capacitive reaction may occur due to the disorder in the interior because of the many internal voids.

In this study, a carbon material featuring these characteristics was synthesized from carbon black for SIBs and very good results were obtained. The carbon material was synthesized using the solution plasma process and the details of synthesis and material analysis are discussed below.

## 2. Materials and Methods

### 2.1. Material Preparation

For the synthesis of carbon black for sodium ion battery (SCB) for SIBs, a process called plasma in solution was used, which has been shown to be a good tool for the synthesis of carbon black [11,12,13,14]. Previous studies developed the synthesis of highly crystalline carbon by using benzene as the carbon precursor. However, in this study, the use of xylene as an aromatic hydrocarbon with two methyl groups enabled the synthesis of hard carbon with low crystallinity when the carbon material was polymerized in the plasma zone. To discharge the plasma in xylene, a tungsten carbide wire with a diameter of 1 mm was wrapped around a ceramic tube (inner diameter: 1 mm, outer diameter: 2 mm), and approximately 1 mm was exposed from the ceramic tube to concentrate the energy at the end. A pair of electrodes was placed in the center of a beaker containing xylene and discharged using a bipolar DC pulsed power supply (Kurita, Kobe, Japan). The experiment was performed at room temperature and atmospheric pressure. The voltage, pulse frequency, and pulse width for plasma generation were controlled as 2.0 kV, 100 kHz, and 1.0 μs, respectively. To improve the electrical conductivity of the synthesized carbon material and remove the hydrogen present inside the carbon, it was heat treated at 500 °C for 3 h under a nitrogen atmosphere in a tube furnace (heating rate: 10 °C/min followed by natural cooling).

### 2.2. Material Characterization

The shape and microstructure of the SCB was observed by High-angle annular dark-field scanning transmission electron microscopy (HAADF-STEM) using a TALOS F200X instrument (Hillsboro, OR, USA) operating at 200 kV. The specific surface area of the SCB was calculated from the N_2_ adsorption-desorption isotherm using the Brunauer–Emmett–Teller (BET) method (Autosorb-iQ, Quantachrome Instruments, Boynton Beach, FL, USA). The sample was pretreated at 200 °C for 2 h to remove moisture before the BET measurements.

### 2.3. Anode Preparation and Electrochemical Test

Electrochemical tests were performed for the SCB using a 2032 coin-type (Wellcos Corp., Royal Oak, MI, USA) half-cell. SCB, a conductive carbon black (TIMCAL Graphite & Carbon Super P), and poly (acrylic acid) (average molecular weight: 3,000,000) binders were mixed in a weight ratio of 7:1:2 and dissolved in distilled water to prepare a slurry. The solution was mixed for 30 min at 400–2000 rpm and 160–800 rpm using an AR-100 conditioning mixer (THINKY Corp., Tokyo, Japan), and then deformed at 2200 rpm for 10 min to produce a slurry. The mixed slurry was uniformly coated on a copper foil using a doctor blade, and the solvent was removed from the slurry by drying in a drying oven at 50 °C for 12 h. It was then compacted to a thickness of 35 μm using a roll press. The mass of the electrode material on the copper foil was about 1.6 mg cm^−2^. The coin cell was assembled in a glove box filled with argon with Na metal as the counter electrode and 1 M NaPF_6_ dissolved in diethylene glycol dimethyl ether (DEGDME) as the electrolyte. The separator was glass filtered and electrochemically tested in the voltage range of 0.005–3 V (vs. Na/Na^+^) using a BCS-805 Biologic battery test system (Biologic, Seyssinet-Pariset, France). The same device was used to perform cyclic voltammetry (CV) to investigate the reduction and oxidation peaks in the voltage range of 0.01–3.0 V (vs. Na/Na^+^) at a scan rate of 0.2 mV/s. Electrochemical impedance spectroscopy (EIS) was performed using a Biologic BCS-805 in the frequency range of 0.01 Hz to 10 kHz with a perturbation amplitude of 10 mV under no-bias conditions. The obtained spectra were fitted with EC-Lab software BT-Lab V1.52 (Bio-Logic Science Instruments, Inc., Seyssinet-Pariset, France).

## 3. Results and Discussion

**Figure 1:**
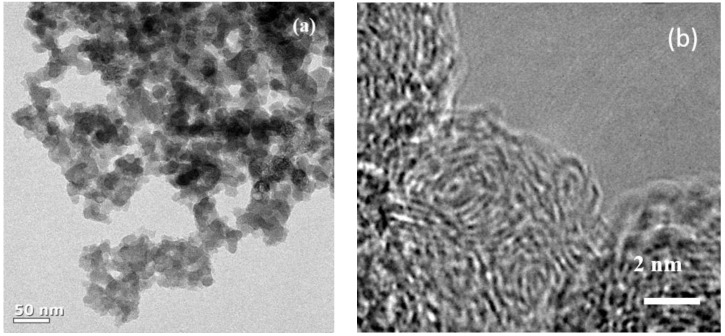
(**a**) Transmission electron microscopy (TEM) and (**b**) High-resolution transmission electron microscopy (HR-TEM) images of the carbon black for sodium ion battery (SCB).

**Figure 2:**
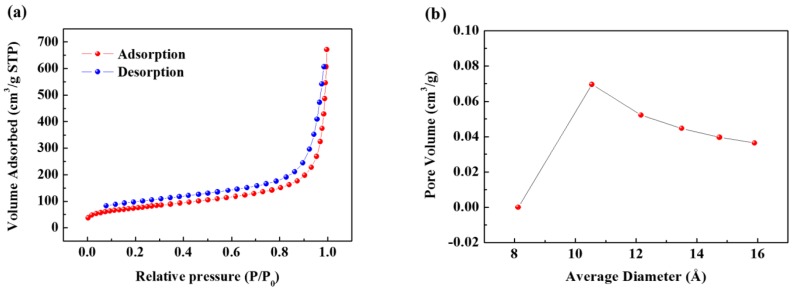
(**a**) Nitrogen adsorption-desorption isotherm curve of the SCB. (**b**) Pore size distribution curve of the SCB.

Figure 1a shows the transmission electron microscopy (TEM) image of the synthesized SCB. As can be seen, spherical primary particles of about 20 to 30 nm size are entangled in different directions, and the aggregate size is at least 1 µm. Figure 1b is a magnified TEM image of the primary particles, which shows that the microcrystalline domains form a turbostratic structure and are oriented in different directions. These domains are composed of only a few layers and have a width of approximately less than 5 nm. Numerous microcrystalline domains of this type aggregated to form spherical primary particle agglomerates, and many voids formed between these domains. Therefore, the aggregate has the structure of carbon black, while the internal structure of the primary particles is almost the same as that of hard carbon. The XRD pattern also shows a peak that is typical of amorphous carbon (Figure S1). Next, N_2_ gas adsorption-desorption curves were obtained by the BET method to evaluate the specific surface area of the SCB (Figure 2a). The specific surface area calculated from the isotherm curve was approximately 268 m^2^ g^−1^. This value is similar to commercial carbon black, and the large specific surface area results from the pore structure that formed on the surface. The pore size distribution (Figure 2b) is calculated from the desorption curve, and it is confirmed that many pores of approximately 10 Å exist on the surface. Therefore, based on the results of TEM and BET, it is confirmed that the SCB is a spherical particle with 10 Å pores on average on its surface and a void structure inside. On the other hand, the calculated planar area of [Na-DEGDME]^+^ was reported to be 7.27 Å × 9.37 Å. Therefore, it is small enough to diffuse into the SCB. In addition, triple complex intercalation of [Na-DEGDME]^+^ is also possible if the distance between graphite layers is wide enough.

**Figure 3:**
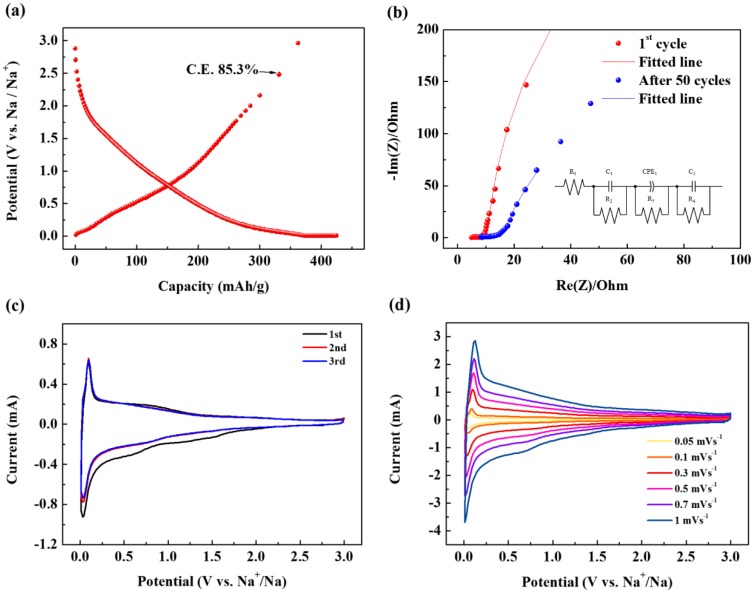

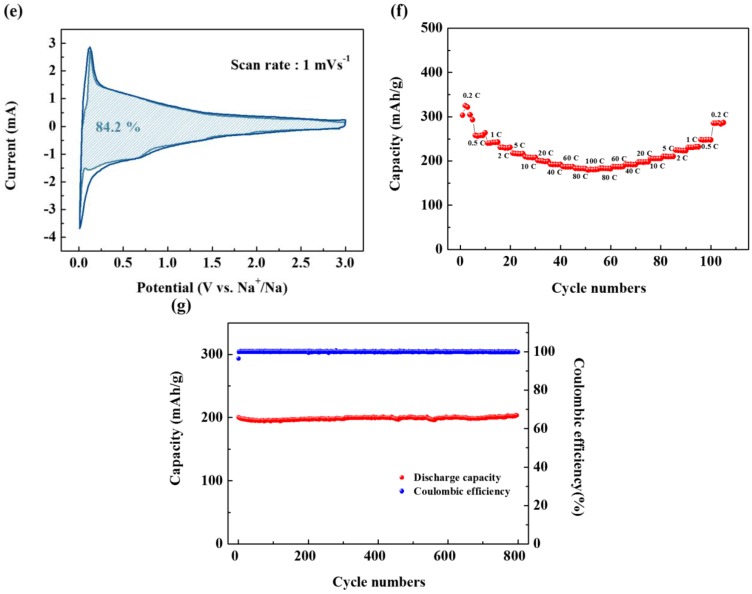
Electrochemical characteristics of SCB: (**a**) Charge-discharge curves of SCB in the first cycle. (**b**) Nyquist plots of the SCB after the 1st and 50th cycles. (**c**) Cyclic voltammogram (CV) curves of the SCB at a scanning rate of 0.2 mV s^−1^ in the voltage range of 0.005–3.0 V (vs. Na/Na^+^). (**d**) CV curves of the SCB at scanning rates of 0.05–1.00 mV s^−1^ in the voltage range of 0.005–3.0 V (vs. Na/Na^+^). (**e**) Capacitive contributions (shaded area) to charge storage at a scanning rate of 1.00 mV s^−1^. (**f**) Rate capability of the SCB. (**g**) Cycling performance of the SCB at a current density of 10 C (350 mA g^−1^).

Next, the electrochemical performance of the SCB was evaluated. Figure 3a represents the first cycle charge/discharge profile of SCB. Here, it is noteworthy that the first cycle C.E. exceeds 85% despite the large surface area of the material. This shows that there is no correlation between the specific surface area and C.E. In fact, graphite with a specific surface area of 1 m^2^ g^−1^, which is 1/330 of the material synthesized in this study, showed 50% C.E. in the ether-based electrolyte via the co-intercalation reaction [5]. In the case of materials with a specific surface area of 100 m^2^ g^−1^, which is one-third of the materials synthesized in this study, the C.E. is extremely low at 55%, even though the same ether-based electrolyte is used [15]. In addition, N330 carbon black with the same carbon black morphology has a specific surface area of only 75 m^2^ g^−1^, but a C.E. of 64%, which is much lower than the SCB [7]. These results indicate that there is no direct relationship between specific surface area and C.E. In other words, co-intercalation cannot occur because a SEI cannot be formed if a wide pathway that can cause a co-intercalation reaction, rather than the specific surface area being well developed. Thus, the surprisingly high efficiency demonstrated in this study can be a strong indicator of the absence of a SEI layer. The absence of a SEI was also evident from the comparison of electrochemical impedance spectroscopy (EIS) measurements as seen in Figure 3b. The depressed nature of the semicircle can be attributed to the merging of the two different semicircles. One is due to the SEI and the other is from the charge-transfer process. Therefore, *R*_1_ means the internal resistance (including the resistance of the electrolyte and electrode) and *R*_2_ corresponds to the resistance of SEI layer while *C*_1_ indicates the resultant capacitance from SEI layer. *R*_3_ is the charge transfer resistance in the intermediate-frequency region and constant phase element (CPE) is related to the surface property of the electrode. *R*_4_ is the Warburg resistance and *C*_2_ indicates the double layer capacitance caused by the ion transfer in the electrode material. The EIS graph in Figure 3b shows that that the resistance of the SEI (*R*_2_) is very small, and even after 50 cycles, it increases very little (Table 1). In particular, this value is much smaller than a SCB discharged in a carbonate-based electrolyte (Figure S2 and Table S1). Therefore, these results are good evidence that no SEI formed on the SCB surface when using the ether-based electrolyte.

Therefore, in this study, the irreversible capacity of approximately 15% in the first cycle is attributed to sodium trapped in some pores in the carbon rather than the SEI formation. When Na is inserted into a soft carbon turbostratic structure, local and macro-structural expansion occurs inside the structure, which is sometimes irreversible. Thus, some Na ions may become trapped in the intercalated site [16]. Figure 3c shows the cyclic voltammogram (CV) of the SCB. The CV clearly shows peaks arising from reversible oxidation and reduction reactions. These peaks appear when Na is stored in the carbonaceous material together with the electrolyte [5]. Thus, the appearance of this peak demonstrates the successful co-intercalation of Na and solvent. On the other hand, in the CV, no distinct peak indicating the decomposition of electrolyte appears, and it can be seen that the 2nd and 3rd peaks are superimposed. This implies that no SEI is formed on the electrode surface, and almost all the Na can be reversibly drawn into the carbon while some amount of Na is trapped.

On the other hand, to investigate the surface dynamics of the electrode reaction, an additional CV analysis at a low scan rate (Figure 3d) was conducted. Generally, in lithium and sodium-ion cells, the CV current is represented by a combination of capacitive and intercalation/deintercalation currents. Therefore, the total charge stored on the electrodes can be distinguished by a combination of capacitive and intercalation mechanisms. According to Power’s law, the voltage-current response of electrode active materials at various sweep rates can be summarized as follows:

*i* = *av*^*b*^ (1)

where *v* is the scan speed (V s^−1^), and d and b are adjustable parameters. The value of *b* can be determined from the slope of the linear fit of the log*i* vs. log*v* plot at a fixed potential (V). At *b* = 0.5, the peak current is linearly related to the square root of the scan rate (*v*^1/2^), which is considered a typical diffusion-controlled lithium storage process. The peak current at *b* = 1 is proportional to the scan speed and indicates surface-controlled charge storage operations [17,18,19]. The plot peak currents (*i*_p_ and *i*_s_) as a function of the scan rate in the logarithmic scale are shown in Figure 3 and Figure S4. Both the curves show a good linear relationship with *R* = 0.98 and a slope of 0.9 (close to 1), suggesting a surface-controlled process such as adsorption. Therefore, most of the current at the peak potential is capacitive. This indicates that the reaction rate is surface controlled and therefore very fast [20]. Based on the dependence between the peak current and scan rate, the contributions of capacitive and intercalation elements can be estimated using the following equations [21,22]:

*i*(V) = *k*_1_(*v*) + *k*_2_(*v*^1/2^) (2)

where *k*_1_(*v*) and *k*_2_(*v*^1/2^) represent the capacitive current and intercalation/deintercalation current respectively, obtained by modifying the above equation.

*i*(V)/*v*^1/2^ = *k*_1_(*v*^1/2^) + *k*_2_ (3)

where *k*_1_ and *k*_2_ correspond to the slope and slice of the linear fitting of the plot of *i*(V)/*v*^1/2^ vs. *v*^1/2^ respectively. If the values of *k*_1_ and *k*_2_ are obtained, the contribution from the intercalation/deintercalation and the capacitive mechanism can be easily distinguished from each potential. The capacitive and diffusion-controlled contributions to the total capacitance are shown in Figure 3e. Figure 3e indicates that capacitive charge storage plays an important role in the overall capacity of the electrode, and the ratio accounts for 84.2% of the total charge storage.

The high contribution of capacitive charge storage is believed to be due to the turbostratic structure of the surface pores formed on the surface of the primary spherical particles. This provides a large electrolyte contact area, thus facilitating the capacitive reaction. Therefore, the CVs obtained at a low scan rate provide evidence that large pores exist on the surface, as predicted by BET. The large pathway due to the turbostratic structure together with this surface pore structure, plays an important role in the capacitive reaction.

On the other hand, these surface structures also provide conditions for excellent rate capability. The rate capability of the SCB is shown in Figure 3f. An initial high capacity of 303 mAh g^−1^ is observed at a current density of 0.2 C after five cycles. On the other hand, when the C-rate was set continuously as 0.2, 0.5, 1, 2, 5, 10, 20, 40, 60, 80, 100, and 0.2 C, reversible capacities of 300, 260, 240, 230, 216, 209, 200, 191, 187, 183, 180 and 286 mAh g^−1^ were obtained. As the current density decreased, the capacity fully recovered. This indicates that the capacitive reaction inside Na-C is stable at various current densities and that the reversible capacity at 0.2 C is 60% of that at 100 C. This implies that even at a high C-rate, the resistance is not large for a charge transfer. Such excellent output characteristics can be achieved only without a SEI. This further reinforces that a SEI was not formed.

Figure 3g shows the cycling performance of the SCB. As can be seen, the SCB shows the same reversible capacity over 1000 cycles, which is about 1.5 times of the previously reported hard carbon [5].

From the above results, it was confirmed that carbon black-type materials with a turbostratic structure (i.e., a wide pathway) containing many voids are highly efficient structures for the storage of Na ions via capacitive reactions. Therefore, for a SIB fabricated using the capacitive reaction, it is possible to produce an excellent anode material for the SIB by considering this structure and the composition with other materials.

**Figure 4:**
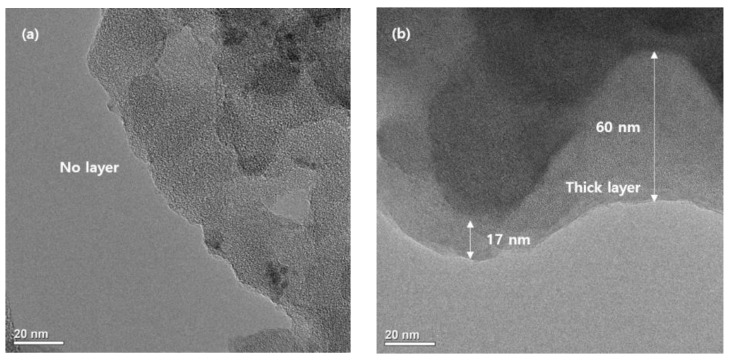
TEM images of the SCB (**a**) discharged in diethylene glycol dimethyl ether (DEGDME) (**b**) discharged in a 1:1 (*v/v*) mixture of ethylene carbonate and dimethyl carbonate.

Finally, a SCB sample was taken from the coin cell after 100 cycles and observed it via TEM. The SCB cycled with the ether-based electrolyte using a carbonate-based electrolyte was also compared (Figure 4). In the case of the SCB taken from the cell using the DEGDME, the SEI layer was not observed, as predicted by the electrochemical analysis. However, in the case of the SCB using the carbonate electrolyte, a thick SEI layer formed on the surface.

## 4. Conclusions

A carbon black material with turbostratic structure using the solution plasma process was successfully synthesized. Very small primary particles of sizes 20 to 30 nm, aggregates of about 1 μm, and a specific surface area of approximately 330 m^2^/g, were obtained. Despite being a nanomaterial, a first cycle coulombic efficiency of 85% for the new material was achieved and confirmed the absence of a SEI through various electrochemical analyses. Based on these results, it was confirmed that an efficient structure can improve the storage capacity of sodium by using the capacitive reaction. Therefore, it is expected that a better sodium storage material can be developed by designing composite materials with other elements based on the structure mentioned in this paper.

## Figures and Tables

**Table 1:** The fitting values for the elements of 1st cycle and after 50 cycles.

	*R*_1_ (Ω cm^−2^)	*C*_1_ (F cm^−2^)	*R*_2_ (Ω cm^−2^)	CPE_1_ (S s^1/2^ cm^−2^)	*R*_3_ (Ω cm^−2^)	*C*_2_ (F cm^−2^)	*R*_4_ (Ω cm^−2^)
1st cycle	4.364	8.903 × 10^−6^	8.714 × 10^−1^	1.281 × 10^−1^	22.29	5.112 × 10^−2^	2063
After 50 cycles	8.488	1.115 × 10^−4^	1.7	5.178 × 10^−2^	10.65	1.179 × 10^−1^	542.2

## Supplementary Materials

The following are available online at https://www.mdpi.com/2079-4991/9/5/793/s1, Figure S1: X-ray diffraction patterns of SCB, Figure S2: Nyquist plots of SCB (in a 1:1 (*v/v*) mixture of ethylene carbonate and dimethyl carbonate) after 1st and 50th cycles, Figure S3: Determination of cathodic peak current values, Figure S4: Cathodic peak current dependence on scanning rate, which is used to determine capacitive and intercalation contributions to energy storage, Table S1: The fitting values for the elements of 1st cycle and after 50 cycles.
