# Supplementary material for: Nano Hard Carbon Anodes for Sodium-Ion Batteries
Source: Nanomaterials (Basel). 2019 May 23;9(5):793. doi: 10.3390/nano9050793 (PMC6567009; doi:10.3390/nano9050793)
Supplement: Supplementary file 1 [file nanomaterials-09-00793-s001.pdf]

Supplementary Information

Nano Hard Carbon Anodes for Sodium-Ion Batteries

Dae-Yeong Kim [1], Dong-Hyun Kim [2], Soo-Hyun Kim [2], Eun-Kyung Lee [3], Sang-Kyun Park [4], Ji-Woong Lee [4], Yong-Sup Yun [5], Si-Young Choi [6,*] and Jun Kang [1,*]

[1] Division of Marine Engineering, Korea Maritime and Ocean University, 727 Taejong-ro, Yeongdo-gu, Busan 49112, Korea; smap1211@kmou.ac.kr

[2] Korea Maritime Equipment Research Institute/ICT Convergence Team, 435 Haeyang-ro, Yeongdo-gu, Busan 49111, Korea; kdh9942@komeri.re.kr (D.-H.K.); shkim@komeri.re.kr (S.-H.K.)

[3] Department of Ocean Advanced Materials Convergence Engineering, Korea Maritime and Ocean University, 727 Taejong-ro, Yeongdo-gu, Busan 49112, Korea; elee@kmou.ac.kr

[4] Division of Marine Information Technology, Korea Maritime and Ocean University, 727 Taejong-ro, Yeongdo-gu, Busan 49112, Korea; skpark@kmou.ac.kr (S.-K.P.); woongsengine@kmou.ac.kr (J.-W.L.)

[5] Division of Marine System Engineering, Korea Maritime and Ocean University, 727 Taejong-ro, Yeongdo-gu, Busan 49112, Korea; ysyun@kmou.ac.kr

[6] Department of Materials Science and Engineering, POSTECH, 77 Cheongam-ro, Pohang 37673, Korea

* Correspondence: junkang@kmou.ac.kr (J.K.); youngchoi@postech.ac.kr (S.-Y.C.); Tel.: +82-51-410-4281 (J.K.); +82-54-279-2161 (S.-Y.C.)

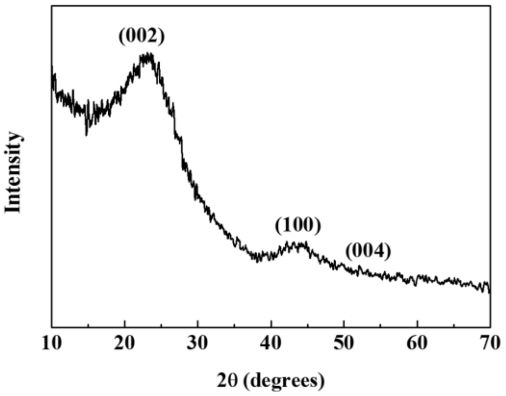

Figure S1. X-ray diffraction patterns of SCB.

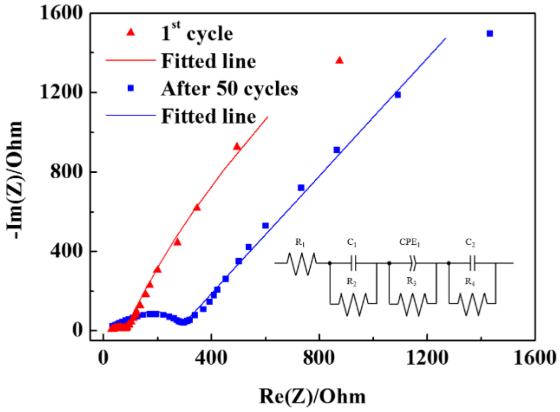

Figure S2. Nyquist plots of SCB (in a 1:1 (v/v) mixture of ethylene carbonate and dimethyl carbonate) after 1st and 50th cycles.

Table S1. The fitting values for the elements of 1st cycle and after 50 cycles.

	R_1 (Ω cm^{-2})	C_1 (F cm^{-2})	R_2 (Ω cm^{-2})	CPE$_1$ (S s$^{1/2}$ cm^{-2})	R_3 (Ω cm^{-2})	C_2 (F cm^{-2})	R_4 (Ω cm^{-2})
1st cycle	24.7	1.193×10^{-1}	55.45	3.804×10^{-4}	66.45	1.109×10^{-2}	2,672
After 50 cycles	16.05	5.161×10^{-3}	241	7.593×10^{-7}	332.7	8.012×10^{-3}	3,285

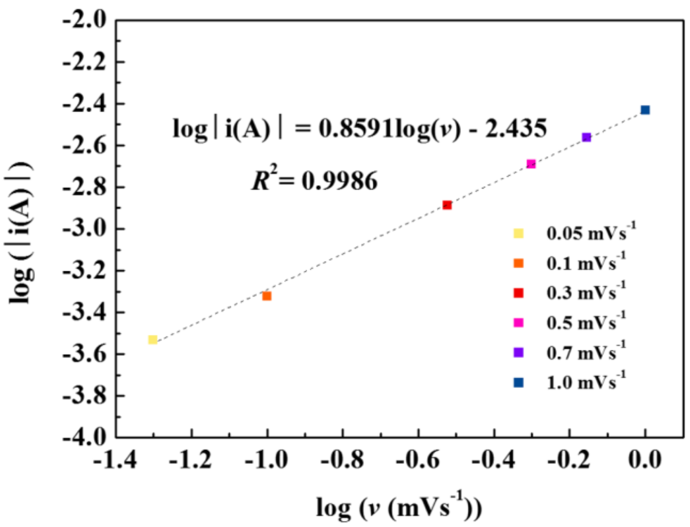

Figure S3. Determination of cathodic peak current values.

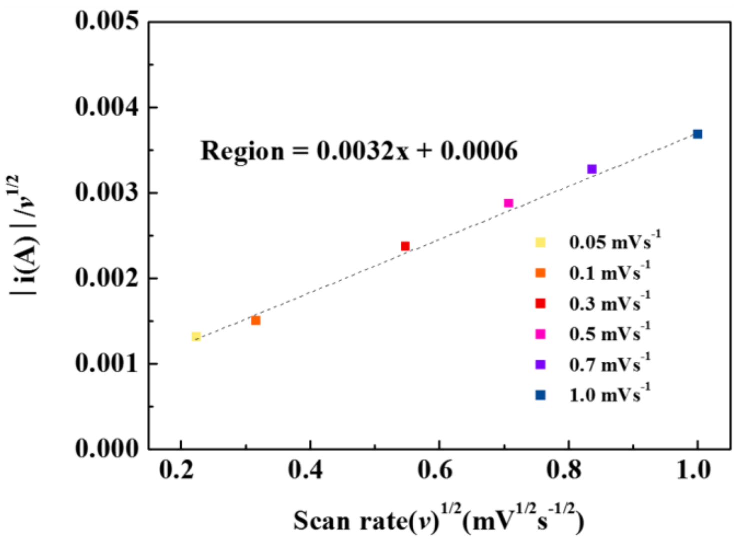

Figure S4. Cathodic peak current dependence on scanning rate, which is used to determine capacitive and intercalation contributions to energy storage.
